# Predictive Place-Cell Sequences for Goal-Finding Emerge from Goal Memory and the Cognitive Map: A Computational Model

**DOI:** 10.3389/fncom.2017.00084

**Published:** 2017-10-12

**Authors:** Lorenz Gönner, Julien Vitay, Fred H. Hamker

**Affiliations:** ^1^Artificial Intelligence, Department of Computer Science, Technische Universität Chemnitz, Chemnitz, Germany; ^2^Bernstein Center Computational Neuroscience, Humboldt-Universität Berlin, Berlin, Germany

**Keywords:** sequential activity, reward-based learning, goal memory, contextual bias, memory recall, continuous attractor network, Bayesian decoding

## Abstract

Hippocampal place-cell sequences observed during awake immobility often represent previous experience, suggesting a role in memory processes. However, recent reports of goals being overrepresented in sequential activity suggest a role in short-term planning, although a detailed understanding of the origins of hippocampal sequential activity and of its functional role is still lacking. In particular, it is unknown which mechanism could support efficient planning by generating place-cell sequences biased toward known goal locations, in an adaptive and constructive fashion. To address these questions, we propose a model of spatial learning and sequence generation as interdependent processes, integrating cortical contextual coding, synaptic plasticity and neuromodulatory mechanisms into a map-based approach. Following goal learning, sequential activity emerges from continuous attractor network dynamics biased by goal memory inputs. We apply Bayesian decoding on the resulting spike trains, allowing a direct comparison with experimental data. Simulations show that this model (1) explains the generation of never-experienced sequence trajectories in familiar environments, without requiring virtual self-motion signals, (2) accounts for the bias in place-cell sequences toward goal locations, (3) highlights their utility in flexible route planning, and (4) provides specific testable predictions.

## 1. Introduction

By their remarkable spatial selectivity, hippocampal place cells have qualified as a model system for studying neural coding in relation to behavior (O'Keefe and Nadel, 1978; Burgess, 2014). Place cells fire when the animal traverses a certain location known as the place field, accompanied by 4–8 Hz theta oscillations in the local field potential (LFP). However, during states of slow-wave sleep and awake resting, hippocampal activity displays brief periods of fast (150–250 Hz) oscillations termed sharp wave-ripple episodes (SWRs). According to the “two-stage” model of memory, SWR events are involved in memory consolidation, facilitating the transfer of labile hippocampal memory traces to neocortical areas (Marr, 1971; Buzsáki, 1989). During these events, place cell activity displays sequential patterns termed forward replay and reverse replay: Time-compressed, and sometimes time-reversed, replicas of place cell activity during previous runs (Skaggs and McNaughton, 1996; Kudrimoti et al., 1999; Diba and Buzsáki, 2007), potentially reflecting the recall of spatial experiences stored in the hippocampus during behavior (Jensen and Lisman, 1996).

Recent research, however, has highlighted several key aspects of SWR-associated sequential hippocampal activity which suggest additional functional roles. It has been demonstrated that the disruption of SWR activity not only impairs spatial learning (Girardeau et al., 2009; Jadhav et al., 2012), but also hinders performance of learned spatial tasks (Jadhav et al., 2012). The depicted trajectories need not be replicas of paths previously traveled (Gupta et al., 2010), and multiple trajectory options can be signaled across SWR episodes (Singer et al., 2013). Furthermore, goal locations are over-represented in place cell activity during SWRs in open-field tasks (Dupret et al., 2010), even in the form of trajectories which predict immediate future behavior (Pfeiffer and Foster, 2013). Consequently, it has been proposed that awake place-cell sequences can guide ongoing behavior by planning future trajectories, particularly toward goal locations (Diba and Buzsáki, 2007; Dupret et al., 2010; Pfeiffer and Foster, 2013; Olafsdóttir et al., 2015) or by evaluating options and decision-making (Carr et al., 2011; Jadhav et al., 2012).

The hypothesis that certain forms of sequential activity can guide behavior implies specific properties of the sequence-generating mechanism. First, for efficient behavioral guidance, sequence trajectories should be task-dependent, depicting currently relevant trajectories preferentially (Singer et al., 2013). Second, trajectories should include novel combinations of start and end points when necessary (Pfeiffer and Foster, 2013). These conditions are not easily met by most existing computational models of sequential hippocampal activity. First, sequence learning models assume that experience-dependent plasticity acts on recurrent synaptic connections in hippoampal area CA3, producing asymmetric, “chain-like” connectivity motifs (Jensen and Lisman, 1996; Redish and Touretzky, 1998; Molter et al., 2007; Bush et al., 2010). In these models, recall sequences emerge which replicate previous experience at a compressed time scale, provided that recurrent synaptic transmission is sufficiently strong (but see Jahnke et al., 2015). A second class of models posits that place-selective subthreshold inputs bias hippocampal place cell activity. Here, a gradual release of inhibition during SWR states causes place cells to activate in the order of the distance between their place field and the current location, generating reverse replay sequences (Foster and Wilson, 2006; Csicsvari et al., 2007; Diba and Buzsáki, 2007). Third, models assuming continuous attractor network dynamics have shown that the incorporation of spike-frequency adaptation or short-term synaptic plasticity leads to a random drift of activity through a spatial map (Hopfield, 2010; Itskov et al., 2011; Azizi et al., 2013; Romani and Tsodyks, 2014). In addition to these phenomenological models, a few approaches have explicitly aimed at generating place-cell sequences with a functional role in goal-directed behavior. A recent proposal is based on linear “look-ahead probe” activity driven by grid cells (Erdem and Hasselmo, 2012; see also Bush et al., 2015; Sanders et al., 2015; Stemmler et al., 2015). While look-ahead models specify how certain possible directions can be evaluated using sequential activity, they do not provide an a priori bias for specific preferred directions. In tasks with a high number of options, such as in open-field navigation, this may result in excessive processing demands (Dolan and Dayan, 2013), unless an additional mechanism specifies the direction toward the goal prior to sequence generation (e.g., Burgess et al., 1994). Using a probabilistic approach, Penny et al. (2013) have shown that goal-predictive sequential activity emerges in a formal model of statistical inference processes. Finally, Corneil and Gerstner (2015) have proposed a model in which a theoretically derived “successor representation” is approximated by a continuous attractor network of non-spiking cells to generate goal-directed sequential activity. However, to the best of our knowledge, there exists as yet no neural-level model that generates sequential activity with a bias toward learned goal locations, with a functional role in guiding behavior, and which is formulated at a sufficient level of detail to allow a quantitative comparison between simulated sequence trajectories and experimental data.

To fill this gap, we present a model of place-cell sequences, implemented in a large-scale spiking network with physiologically interpretable parameters, in which goal learning by reward-based plasticity shapes the sequence generation process, and in which sequential activity guides spatial behavior. In our model, following reward-based potentiation of cortico-hippocampal synapses, prefrontal contextual representations bias hippocampal recall activity, which progresses sequentially across the cognitive map-like network structure toward a context-specific goal location. Importantly, sequence trajectories neither replicate previous experiences nor follow virtual directional signals, but rather emerge as an effect of intrinsic network dynamics biased by goal-specific inputs. The resulting place-cell sequences, in particular their end points, are used to guide the behavior of a virtual rat in a memory-guided decision-making task. Furthermore, the implementation as a large spiking network showing ripple-band oscillations allows to employ a Bayesian decoding approach, as used in experimental studies, and to analyse the dynamics of emerging sequential place representations in detail.

## 2. Materials and methods

### 2.1. Model architecture

**Figure 1:**
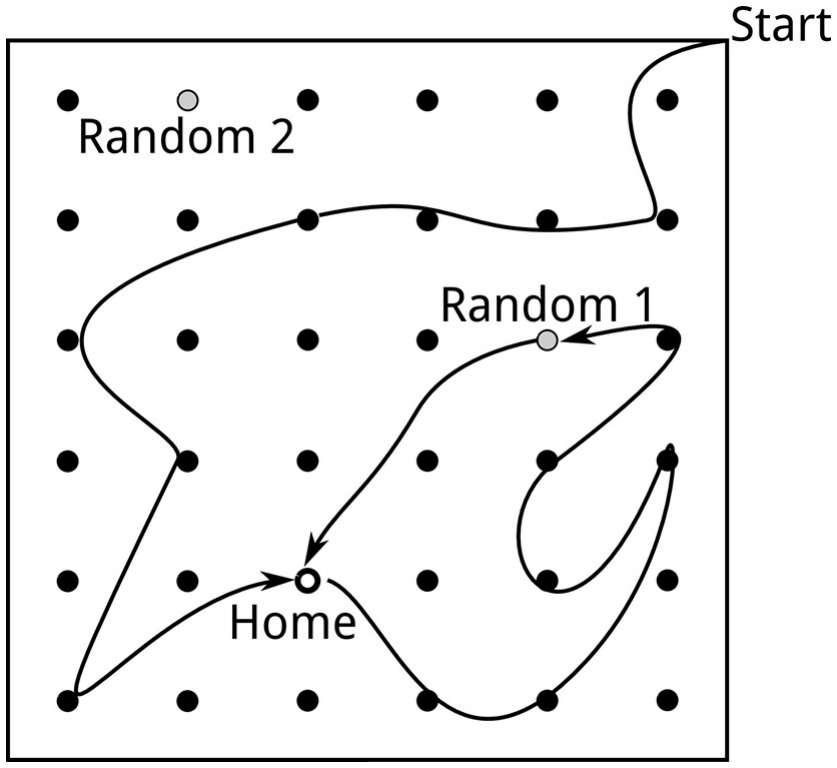
Task setup as described by Pfeiffer and Foster (2013). A square arena (2 m × 2 m) is equipped with 36 potential reward locations. The location first baited with reward is called Home. When the Home location is discovered, the next reward will be placed at a random location, followed by the Home location, etc. A trial consists of the rat approaching the Home location and then foraging until the next random reward is found.

**Figure 2:**
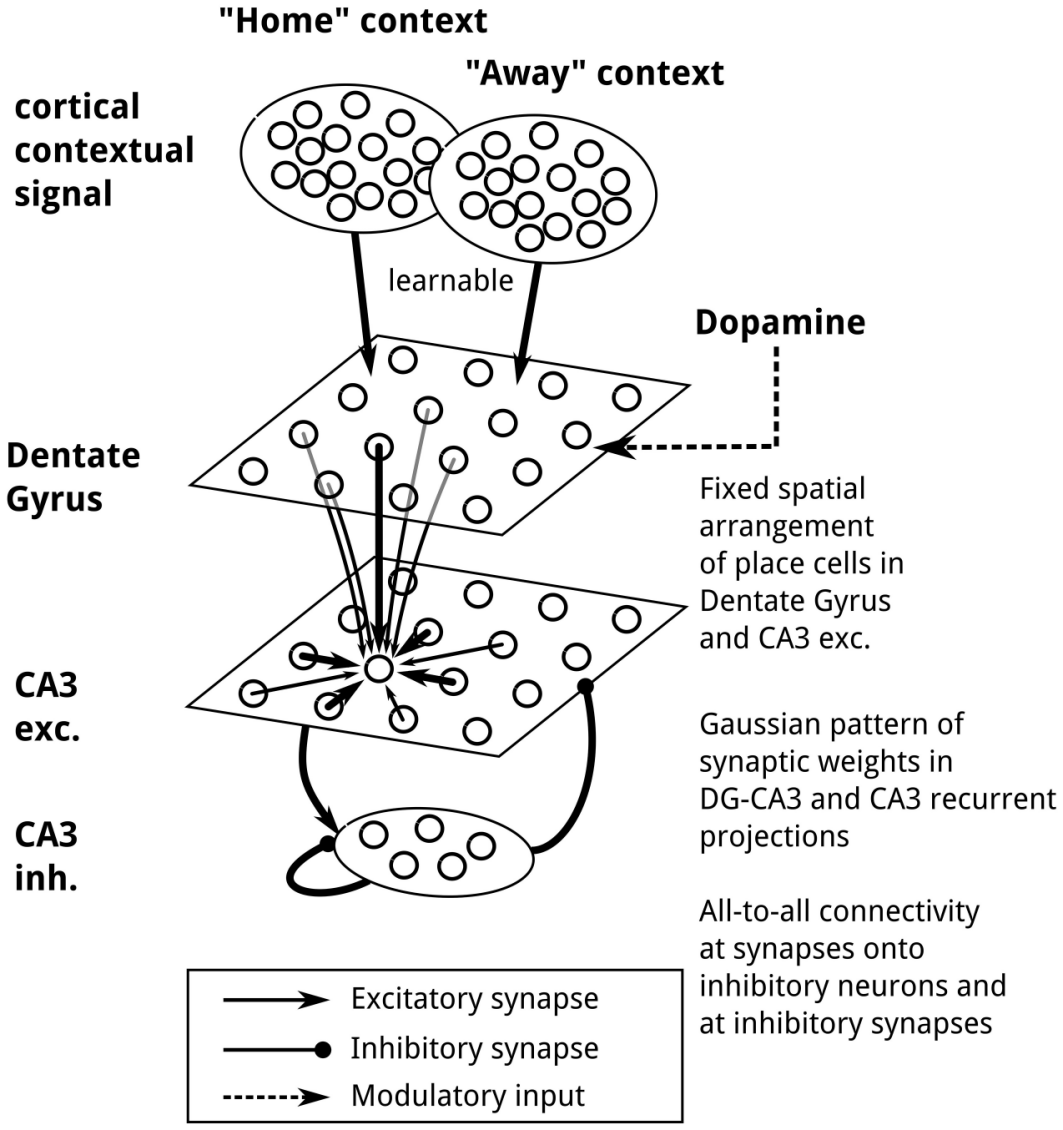
Network architecture. Two context populations of cortical cells project onto model DG granule cells, with connections modifiable by reward-dependent Hebbian plasticity. DG and CA3 cells are spatially arranged on a regular lattice, ordered by the position of place field centers. Connection weights between DG and CA3 place cells and between CA3 cells follow a Gaussian function of distance. CA3 place cells project to an inhibitory population featuring recurrent inhibitory connections and projecting back to CA3 place cells. During movement, place field activity in DG and CA3 cells is generated by external stimulation, and recurrent synaptic transmission is inactivated. Cortical “Home” context population, 6,400 neurons; cortical “Away” context population, 6,400 neurons; DG population, 6,400 neurons; CA3 excitatory population, 6,400 neurons; CA3 inhibitory population, 259 neurons.

We implemented a network-level model of context-dependent learning and recall of goal locations, capable of guiding a virtual rat in a memory-guided decision-making task in which navigation toward a familiar reward location alternates with random foraging (Pfeiffer and Foster, 2013, see Figure 1). Two key properties of the model are that (1) following the learning of a reward location, goal-directed place-cell sequences will be generated, and (2) the end points of these sequences guide subsequent navigational behavior, in a manner sensitive to the current behavioral context. Conceptually, our model network consists of a contextual layer, inspired by prefrontal cortical areas, and a simplified hippocampus model, whose populations represent the dentate gyrus (DG) and subfield CA3. The contextual layer contains two separate populations to reflect the two-phase structure of the simulated task, which we assume has been learned already, as the experimental data reported from this task were recorded from well-trained animals. The activity of these two populations, termed “Home” and “Away” context, indicates whether the current task is to find the familiar reward location called “Home” or to forage randomly for reward. Note that contextual coding has been observed in prefrontal cortical areas (Hyman et al., 2012; Waskom et al., 2014; Long and Kahana, 2015; Rossato et al., 2015; Ma et al., 2016; see also Benoit et al., 2014). The hippocampus model consists of place cells in DG and CA3, as well as inhibitory interneurons in CA3 (see Figure 2; for an anatomical review of the hippocampal formation, see Amaral and Lavenex, 2006). Reward-based plasticity is implemented at context-to-DG synapses to implement learning of context-specific goal locations.

In short, the functioning of our model relies on recall activity in CA3 place cells, biased by the cortico-DG pathway, at which information about goal locations and context (in the sense of task phase) converge. CA3 is configured as a continuous attractor network model, displaying “bump” activity states which will either persist at one location, move to neighboring locations gradually, or transition to a distant location abruptly, depending on the spatial activity profile of its inputs relative to its current activity peak (Ben-Yishai et al., 1995; Degris et al., 2004; Song and Wang, 2005; Fung et al., 2008).

In our simulations, we distinguish between two dynamical states of neural activity during behavior of a virtual rat in a 2 m × 2 m square environment. Our simulations encompassed place field activity during movement and “off-line” activity of place cells during brief pauses in behavior, assumed to occur at the beginning of each run. When the simulated animal is stationary (i.e., at the start of the trial and following discovery of reward), CA3 recurrent transmission is activated, with synaptic weights forming a pattern of short-range excitation and global inhibition, to produce continuous attractor dynamics. During movement, we generate place field activity in DG and CA3 place cells by injecting an external current that varies as a function of the location of the simulated animal. To speed up simulations, and in accord with several previous models, we assume that CA3 recurrent transmission during movement phases is negligible (Molter et al., 2007; Bush et al., 2010; Gupta, 2011). We refer to these two network states as sequence generation and movement states.

### 2.2. Neuron model

DG and CA3 cells are represented by a leaky integrate-and-fire model with parameters similar to a standard excitatory neuron (Brette and Gerstner, 2005). Context cell firing is modeled as a Poisson process, with firing rates during active epochs at either 10 Hz (during simulated movement) or 200 Hz (during sequence generation). The membrane potential *u*_DG_ of DG granule cells is subject to excitatory currents *I*_exc_ from context cell inputs and a place-specific current injection *I*_ext_ (see below):

$$\begin{array}{l} C\frac{du_{\text{DG}}}{dt}=-g_{L}\left(u_{\text{DG}}-E_{L}\right)+I_{\text{exc}}+I_{\text{ext}}. \end{array}$$

CA3 excitatory and inhibitory cells contain an additional inhibitory synaptic current *I*_inh_:

$$\begin{array}{l} C\frac{du_{\text{CA3}}}{dt}=-g_{L}\left(u_{\text{CA3}}-E_{L}\right)+I_{\text{exc}}-I_{\text{inh}}+I_{\text{ext}}+C\cdot \xi . \end{array}$$

Here, ξ is a random variable drawn from a Gaussian distribution with zero mean and 2 mV/ms standard deviation, which serves as background input to the CA3 attractor network, *g*_*L*_ = 30 nS, and *C* = 300 pF. CA3 excitatory and inhibitory cells have an absolute refractory period of *t*_refr, exc_ = 3 ms and *t*_refr, inh_ = 4 ms. The after-spike reset value is *E*_*L*_ = −70.6 mV for all populations.

### 2.3. Network layout, topology, and connectivity

The 6,400 DG cells and 6,400 CA3 cells were arranged on a regular 80 × 80 grid, ordered by their place field centers. To facilitate display of weight matrices, each of the 6,400 neurons of the “Home” and the “Away” context population projected to a single DG neuron. The strength of synapses from DG to CA3 excitatory cells and at CA3 recurrent excitatory synapses follows a Gaussian connectivity pattern, resulting in strong local connectivity. The projections to, from, and among CA3 inhibitory neurons are all-to-all with uniform synaptic strengths within each projection (for exact values, see Table 1).

**Table 1:** Network connectivity.

**Synapse group**	**Connectivity**	**Weight type**	**Weight**
“Home” context to	One-to-one	Learnable	Initial:
DG		Random uniform initialization	[0 …0.3 nA]
“Away” context to	One-to-one	Learnable	Initial:
DG		Random uniform initialization	[0 …0.3 nA]
DG to CA3 exc.	Local, within	Gaussian profile,	6.6 nA (max.)
	50 cm distance	σ_DG-CA3_ = 50 cm, fixed	
CA3 exc. recurrent	All-to-all,	Gaussian profile,	0.82 nA (max.)
	no autapses	σ_CA3_ = 50 cm,	
		fixed, inactive during	
		simulated rat movement	
CA3 exc. to CA3	All-to-all	Uniform, fixed, inactive	9.75 pA
interneurons		during running	
CA3 interneurons	All-to-all	Uniform, fixed	0.468 nA
recurrent			
CA3 interneurons	All-to-all	Uniform, fixed	18.45 pA
to CA3 exc.			

For consistency with spatial learning, a bounded network topology was chosen. To avoid edge effects, the 80 × 80 network grid was identified with a virtual environment extending beyond the simulated arena. As pilot simulations indicated that smaller sizes of the attractor bump were accompanied by very high firing rates of CA3 excitatory cells, less consistent with experimental data, we chose network parameters that resulted in a broader bump with lower individual firing rates. Therefore, for the simulation of the task used by Pfeiffer and Foster (2013), the entire network was identified with a virtual environment size of 4.2 m × 4.2 m, of which only an interior section of 2 m × 2 m could be visited by the simulated rat.

### 2.4. Synapses

Current-based synapses were used with instantaneous rise and exponential decay:

$$\begin{array}{l} \tau_{\left\{\text{exc},\text{inh}\right\}}\frac{dI_{\left\{\text{exc},\text{inh}\right\}}}{dt}=-I_{\left\{\text{exc},\text{inh}\right\}}, \end{array}$$

where τ_exc_ = 6 ms, and τ_inh_ = 2 ms. Following recent proposals for the generation of sharp wave-ripple oscillations by recurrent inhibition (Schlingloff et al., 2014; Stark et al., 2014), connections between and within both CA3 populations had a uniform 2.5 ms delay.

### 2.5. Place fields

Place-specific firing in DG granule cells and CA3 pyramidal cells was generated by external stimulation:

$$\begin{array}{l} I_{\text{ext},j}\left(t\right)=I_{\text{max}}\exp\left(-\frac{{\left(x-x_{j}\right)}^{2}}{\sigma_{\text{PF}}^{2}}\right), \end{array}$$

where *x* is the simulated animal's current location and *x*_*j*_ is the place field center, *I*_max_ = 10 nA and σ_PF_ = 25 cm.

### 2.6. Synaptic plasticity

Learning at the synapses from context cells onto DG granule cells requires pre- and postsynaptic activity and the presence of a reward-related signal such as a transient increase or decrease in postsynaptic dopamine, which has been shown to modulate the plasticity of DG input synapses (Manahan-Vaughan and Kulla, 2003). We assumed a simplified phasic reward signal in DG granule cells. This signal takes a value of 1 immediately when the simulated rat finds reward, or −1 when it does not find reward at a position where it searched for it (for details of the behavioral simulation, see subsection “Simulated Task”). After 100 ms, the reward signal is reset to zero. The weight change is given by:

$$\begin{array}{l} \frac{dw_{ij}}{dt}=\alpha_{L}{\left[{\bar{x}}_{i}\left(t\right){\bar{y}}_{j}\left(t\right)-w_{ij}\right]}^{+}\;\text{if }R_{j}=1,\text{ and}\\ \frac{dw_{ij}}{dt}=-\alpha_{L}{\bar{x}}_{i}\left(t\right){\bar{y}}_{j}\left(t\right)\;\text{if }R_{j}=-1, \end{array}$$

where ${\bar{x}}_{i}$ and ${\bar{y}}_{j}$ denote pre- and postsynaptic activity traces, which are updated whenever the respective neuron spikes:

$$\begin{array}{l} \tau_{\text{trace}}\frac{d\bar{x}}{dt}=-\bar{x}\\ \bar{x}\left(t\right)=1\;\text{if }t=t_{\text{spike}}. \end{array}$$

*R*_*j*_ indicates the postsynaptic reward signal, α_*L*_ = 50 nA/s is the learning rate, τ_trace_ = 100 ms, and [*x*]^+^ = max(*x*, 0). This form of learning rule avoids the problem termed “occupancy bias”: Standard Hebbian learning would lead to repeated potentiation every time a rewarded location was visited, creating a dependency of weight strength on the number of visits to that location (Csizmadia and Muller, 2008). For related approaches, see Redish and Touretzky (1998); Lisman and Otmakhova (2001); Csizmadia and Muller (2008); Vitay and Hamker (2010).

### 2.7. Data analysis

During sequence generation embedded in behavioral simulations, the activity bump's center of mass was computed in a sliding window of 4 ms length. Additionally, to compare different network parameter settings under identical conditions, we generated sequences for all network configurations using context-to-DG weight matrices obtained during behavioral simulations. During these analyses, we recorded spiking activity in time frames of 5 ms length, advanced in increments of 2 ms. For each frame, we decoded the location represented by spiking activity using a Bayesian decoding method used in previous experimental studies (Davidson et al., 2009; Pfeiffer and Foster, 2013). The posterior probability of the location *X* represented in neural activity to be a potential location *x* out of a set of position bins ${\left\{x_{j}\right\}}_{j=1}^{M}$, given an observation $r={\left\{r_{i}\right\}}_{i=1}^{N}$ of neural activity *R*, is:

(1) $$\begin{array}{r} P\left[X=x|R=r\right]=\frac{\left(\overset{N}{\underset{i=1}{\prod }}f_{i}{\left(x\right)}^{r_{i}}\right)e^{-\tau \overset{N}{\underset{i=1}{\sum }}f_{i}\left(x\right)}}{\overset{M}{\underset{j=1}{\sum }}\left(\overset{N}{\underset{i=1}{\prod }}f_{i}{\left(x_{j}\right)}^{r_{i}}\right)e^{-\tau \overset{N}{\underset{i=1}{\sum }}f_{i}\left(x_{j}\right)}}, \end{array}$$

where *f*_*i*_ is the spatial tuning curve of unit *i*, *r*_*i*_ is its spike count, and τ is the length of the decoding window. This approach assumes that all *N* units follow independent Poisson firing statistics, and that occupancy is uniform across locations (Davidson et al., 2009). Although we have not examined the degree to which network activity matches the assumption of independent firing, we verified that the Bayesian estimates were highly similar to the results obtained from a population vector decoding scheme (data not shown). The maximum number of cells from which we could simultaneously decode using Equation (1) varied between approximately 200 and 500 cells depending on decoding bin size and activity patterns. Larger sample sizes resulted in all-zero posterior probability distributions, likely owing to the numerical inaccuracies caused by multiplying large numbers of near-zero values in the decoding formula (Leibold, 2011). We therefore subdivided the network randomly into 40 subsets of 160 cells each and performed Bayesian decoding on each subset independently, with a spatial bin size of 2.625 cm. For each subset, position estimates per frame were determined as the center of mass of the posterior probability distribution. For display, posterior probability distributions were summed across time. Additionally, for the display in **Figure 5**, we averaged across the 40 resulting posterior probability distributions, and obtained position estimates from the resulting mean values.

To discriminate between jump-like and gradual movement of the activity bump, we used two different criteria: First, we determined the bump movement per frame as the Euclidean distance between the locations decoded from consecutive frames. Following previous experimental studies, sequential events in which the maximum movement per frame exceeded a certain threshold were classified as jump-like (Pfeiffer and Foster, 2013, 2015), with a threshold value of 40 cm. As an additional criterion, we applied the mean shift clustering algorithm (Comaniciu and Meer, 2002) to detect the number and locations of local maxima in the spatial distribution of spiking activity across the network sheet, with an adaptive bandwidth parameter (default value 52.5 cm). The first 50 ms of each simulated sequence, during which the attractor bump formed at its initial location, were excluded from this analysis.

### 2.8. Simulation environment

The full model was implemented using the Brian simulator, version 1.4.1 (Goodman and Brette, 2009). As all differential equations in the model are linear, exact integration was used, with an integration step of 0.2 ms. For additional analyses comparing different network parameter settings, the sequence generation component of the model was implemented using the ANNarchy simulator, version 4.5 (Vitay et al., 2015). Code will be published in the ModelDB database following publication (http://senselab.med.yale.edu/ModelDB/).

### 2.9. Simulated task

We simulate both neural activity and rat behavior during the spatial learning task described by Pfeiffer and Foster (2013). At the start of a block of trials, the virtual rat is placed at random in one of the corners of the 2 m × 2 m simulated square arena (Figure 1). During the first trial, the rat has to search for the “Home” reward location, which remains fixed during the entire block of trials. “Home” locations are counterbalanced across networks. “Home” trials alternate with “Random” trials, in which the simulated reward is delivered at a random well.

**Figure 3:**
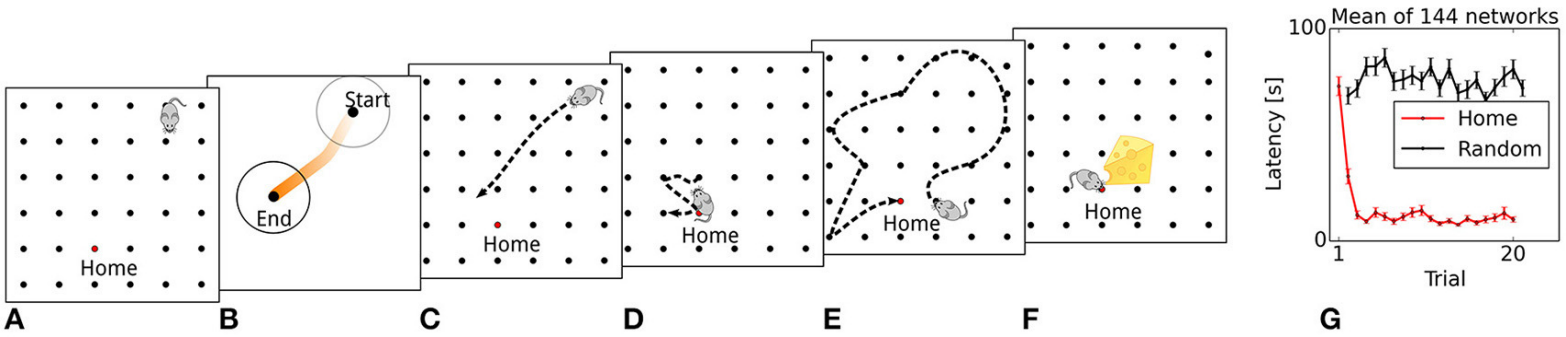
Simulated rat behavior and behavioral performance. **(A)** The virtual rat's physical location at the start of the trial. **(B)** A sequence is generated that originates at the rat's current location. **(C)** The simulated rat navigates toward the location depicted by the sequence end point. **(D)** A focal search is performed around the location defined by the sequence end point. **(E)** Random search until reward is found. **(F)** A modulatory signal is triggered by reward. **(G)** Reward latencies across trials, mean ± s.e.m. Reward latencies in Home trial phases decrease sharply after the first trial, indicating that the simulated rat takes a short path to the Home location from the second trial on.

The time course of simulations during a single trial can be summarized as follows: At the beginning of each trial, the context population that corresponds to the trial type is activated, with a Poisson activity of 10 Hz. A single contextually biased place-cell sequence is initiated, followed by navigation toward the location associated with the end point of the sequence (Figures 3A–F). Generation of a sequence involves activating the CA3 recurrent excitatory synapses and initializing the attractor network by injecting a place-specific external current into CA3 place cells, so that an activity bump representing the current location emerges within 50 ms. Next, the “context population” firing rates are increased to 200 Hz, consistent with the hypothesis that cortical excitatory drive can shape replay activity (Battaglia et al., 2011). After another 350 ms, the location of the bump center at the end of the sequence generation phase is taken as the next navigational goal, which the virtual rat then approaches in a vector-based fashion.

Movement is executed in steps of 100 ms at a constant speed of 15 cm/s, with noise drawn from a Gaussian distribution with zero mean and 0.5 cm variance added to the *x* and *y* components of the movement vector during every motion step. During navigation, DG and CA3 place cells receive a place-specific external stimulation current when the rat's position overlaps their place field. At the same time, the recurrent connections between CA3 place cells are inactivated to reduce the computational load. (Note that this does not affect simulation results: The reduced activity of context populations ensures that DG and CA3 activity signals only the current location, but not the goal, during navigation. Further, synaptic plasticity at the context-to-DG projection is not affected by CA3 activity levels. Finally, model CA3 neurons as well as synapses do not contain any variables depending on spike history, which means that the model dynamics during sequence generation are fully determined by the initial bump location and the cortex-to-DG weight matrices). If the simulated animal moves within 5 cm of the currently baited location, reward is assumed to be found. To model the effect of dopaminergic influence, a reward-related signal in DG granule cells is set to a value of 1 for 100 ms and then reset to zero to transiently enable long-term plasticity at the lateral perforant path synapses.

If the virtual rat does not encounter the active reward location when visiting the place defined by the last sequence's end point, it performs a focal search around that place, similar to the search behavior of mice in the Morris water maze (Ruediger et al., 2012), visiting the four feeder locations nearest to the location defined by the sequence end point. Ultimately, random exploration is performed until the reward location is found, in a form of directed search that will eventually probe all feeder locations exactly once. If reward is not found near the location signaled by the previous sequence end point, the reward signal is immediately set to a value of −1 for 100 ms.

## 3. Results

### 3.1. Behavioral performance

We first evaluate the model's behavioral performance in the simulated memory-guided navigation task described by Pfeiffer and Foster (2013). Across all trials, reward latencies in Home trial phases are substantially lower on average than in Away trial phases (14.8 vs. 74.8 s), demonstrating that the model learns and uses the spatio-temporal reward contingencies. The temporal evolution of reward latencies shows that the mean duration to reach the Home reward location decreases sharply after initially visiting the Home location in the first trial (Figure 3G), consistent with the behavioral results reported by Pfeiffer and Foster (2013). This one-shot learning pattern is similar to the rapid hippocampus-dependent re-learning of goal locations within familiar environments after a contingency switch (Steele and Morris, 1999). We next describe the generation and development of place-cell sequences, resulting from memory recall of learned goal locations, in more detail.

### 3.2. Goal encoding by reward-based plasticity of context-to-DG synapses

**Figure 4:**
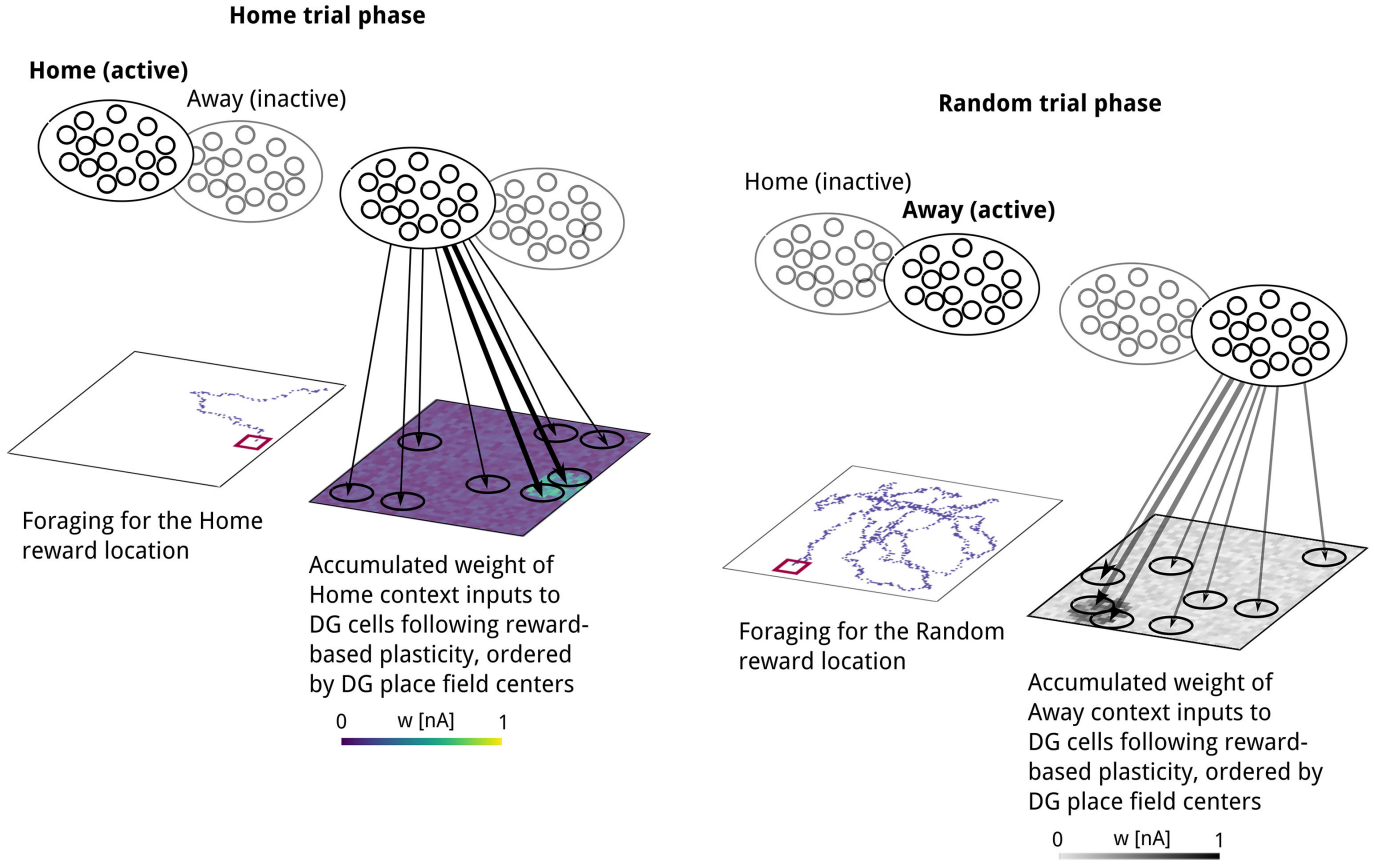
Illustration of context-specific spatial learning, based on simulation data. Reward-dependent Hebbian plasticity potentiates synapses between the currently active context population and DG cells with place fields near the reward location. **Left:** Movement trajectory and resulting pattern of weights between “Home” context cells and DG after discovering the reward location in a “Home” trial phase. **Right:** Movement trajectory and resulting pattern of weights between Away context cells and DG after discovering the reward location in a Random trial phase. As weight changes in a given trial occur at only one of the two projections originating at “Home” or “Away” context cells, we used different color maps to emphasize this contextual selectivity in learning. Line width illustrates the accumulated connection strength schematically.

In our model of spatial learning, the strength of context-to-DG synapses is modifiable by reward-modulated plasticity, implementing a form of goal memory (Seidenbecher et al., 1997); see Figure 4 for illustration. Consequently, lasting changes in synaptic efficacy will lead to a differentiation of post-synaptic DG activity during periods of increased context population activity, potentially biasing hippocampal activity during SWRs.

**Figure 5:**
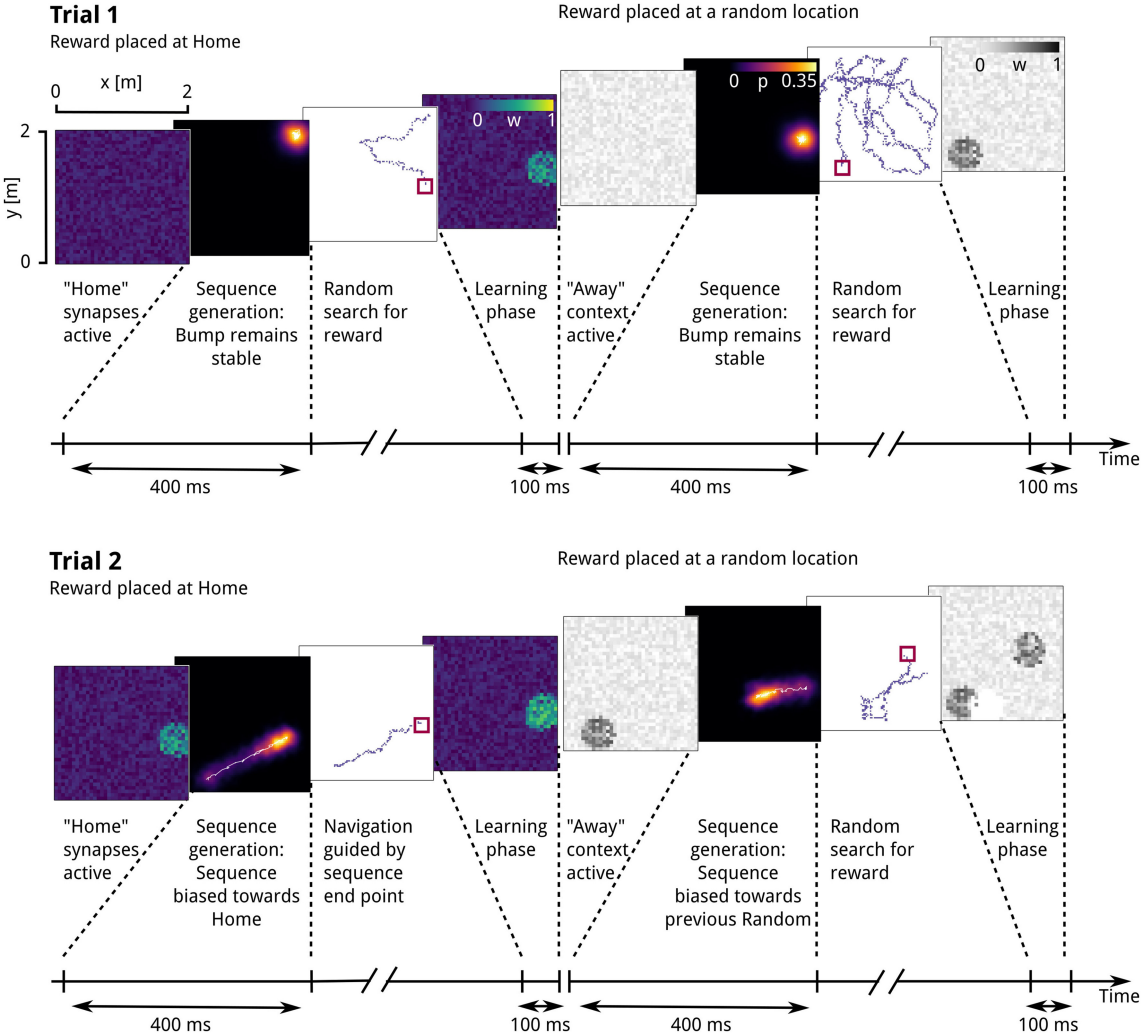
Development of synaptic weights, sequential activity and goal-directed behavior. Time course of the first two simulated trials showing the evolution of context-to-DG synaptic weights, place-cell sequences and behavior. From left to right: Context-to-DG weight matrix at the start of the Home trial phase, decoded sequence trajectory, movement trajectory, context-to-DG weight matrix at the end of the Home trial phase, and same for Away trial phase. Each synaptic weight value is plotted at the location of its corresponding postsynaptic place field center. In the first Home trial **(top left)**, the activity bump persists at the rat's starting location (lower right corner) as no information about the goal location is encoded in Home context-to-DG synapses. The simulated rat therefore performs a random search until the Home reward location is found, triggering synaptic plasticity. A similar pattern is repeated in the first Random trial phase **(top right)**. At the start of the second Home trial **(left center)**, Home context-to-DG synapses encode information about the goal location, sufficient to bias the attractor network bump to move toward the Home location, creating sequential activity. The virtual rat finds reward by navigating toward the location signaled by the sequence end point. Note that the sequence trajectory is a novel path not previously visited by the virtual rat. In the second Random trial phase **(right center)**, the corresponding place-cell sequence guides the rat toward the previous Random reward location, which is now inactive, leading to a weight decrease, and followed by random foraging for the next reward.

As a result of reward-based learning, a weight pattern emerges at context-to-DG synapses that reflects the distance between the post-synaptic cell's place field and the reward location. Weights were initially assigned a low random value. We used a Hebbian learning rule with an eligibility trace, gated by the presence of a reward-related signal, so that a positive reward signal led to long-term potentiation and a negative reward signal induced long-term depression of weights. Following the first visit to the Home reward location, synapses between the Home context population and DG neurons show stronger weights onto DG neurons with place fields closer to the reward location (Figure 4, left). Searching for reward at a non-rewarded location caused weights at synapses onto recently active place cells to decrease considerably (Figure 5, bottom right), indicating that this type of plasticity supports rapid relearning.

To summarize, our learning rule led to increased strengthening of context-to-DG synapses onto DG cells with place fields closer to a location persistently paired with reward, and led to rapid weakening of synapses in the case of unexpected reward omission.

### 3.3. Generation of goal-anticipating place-cell sequences

**Figure 6:**
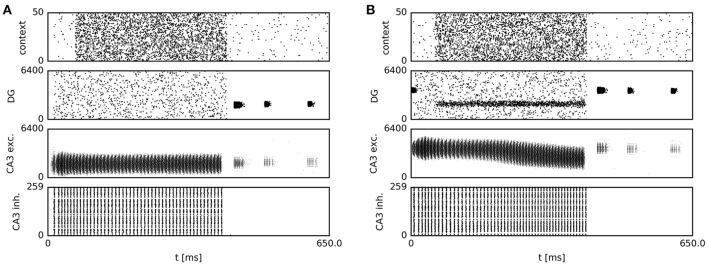
Spiking activity during sequence generation and subsequent navigation. Prior to sequence onset, recurrent transmission is switched on, and a place-specific external current is delivered briefly to CA3 place cells. During this period, context cells fire at 10 Hz, and recurrent dynamics are dominant in CA3. After 50 ms, when the current location is reliably represented by the CA3 network activity bump, context firing rates are increased. The resulting increase in feed-forward excitation via context-to-DG and DG-CA3 synapses causes the onset of sequence generation. Once sequential activity has terminated, navigation continues, accompanied by place cell activity in DG and CA3. **(A)** Persistent CA3 activity in the presence of homogeneous input before learning. **(B)** As a result of increased context drive, and following reward-dependent learning, model DG cells with place fields near the reward location fire at elevated rates, providing a spatial bias to the CA3 continuous attractor network. In response to this bias, the CA3 activity bump gradually centers on those cells with place fields near the reward location. As the activity of cortical context cells is homogeneous across the population, only a subset of 50 cells is shown for clarity of display.

We next describe the effect of potentiated context-to-DG weights on the temporal evolution of network activity. At the beginning of the first Home and Random trials, when input synapses remained in the weak, homogeneous initial state before the onset of contextual goal memory formation, the activity bump of CA3 neurons persisted at the location where it was initialized, corresponding to the virtual rat's current position (Figure 5, top left). A raster plot of the underlying spiking activity is shown in Figure 6A. The simulated rat therefore performed a random search strategy until reward was found, inducing reward-based potentiation of synapses between the “Home” context population and DG cells with a place field near the Home location. A similar pattern is repeated during the first Away trial phase, but with modifications occurring at the synapses between “Away” context cells and DG place cells (Figure 5, top right).

**Figure 7:**
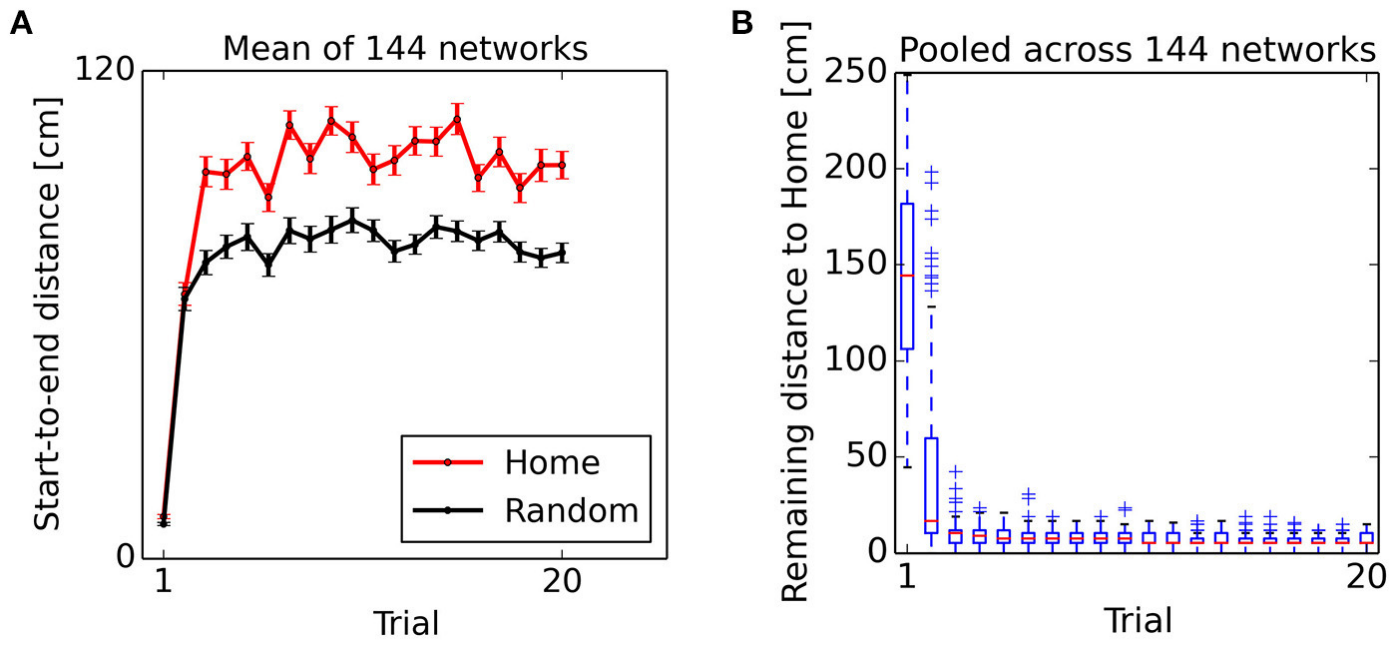
Onset of goal representation. **(A)** Start-to-end distance of sequences, mean ± s.e.m. Bump movement is negligible in the first Home and Random trial phases when no information about the reward location has been encoded in context-to-DG synapses. From the second trial on, sequence trajectories span a considerable distance. **(B)** Remaining distance between sequence endpoint and reward location during Home trial phases. The Home reward location is represented with high accuracy from the second Home trial on. Data pooled across 144 networks. Lower end, red line and upper end of the box show lower quartile, median and upper quartile. Whiskers extend up to 1.5 times the interquartile range (IQR). Crosses denote points extending more than 1.5 times IQR beyond the median.

From the second “Home” trial on, alterations in synaptic strength at “Home” context-to-DG synapses caused substantial heterogeneity in DG firing rates (Figure 6B), sufficient to disrupt the stability of the initial activity state of the CA3 continuous attractor network. As a result, the bump center gradually moved toward those cells receiving maximum input, associated with place fields near the reward location (Figure 5, bottom left). For an overview of the first two Home and Away trial simulations, see Supplemental Video 1. The development of place-cell sequence trajectories shows a sudden onset in the second trial, as can be seen in the time course of distance traveled by the attractor bump (Figure 7A). The accuracy with which the Home location is represented in population activity at the end of the sequence converges rapidly, with a remaining mean error of approx. 10–15 cm (Figure 7B), well sufficient to disambiguate reward locations separated by approx. 33 cm in the virtual maze.

**Figure 8:**
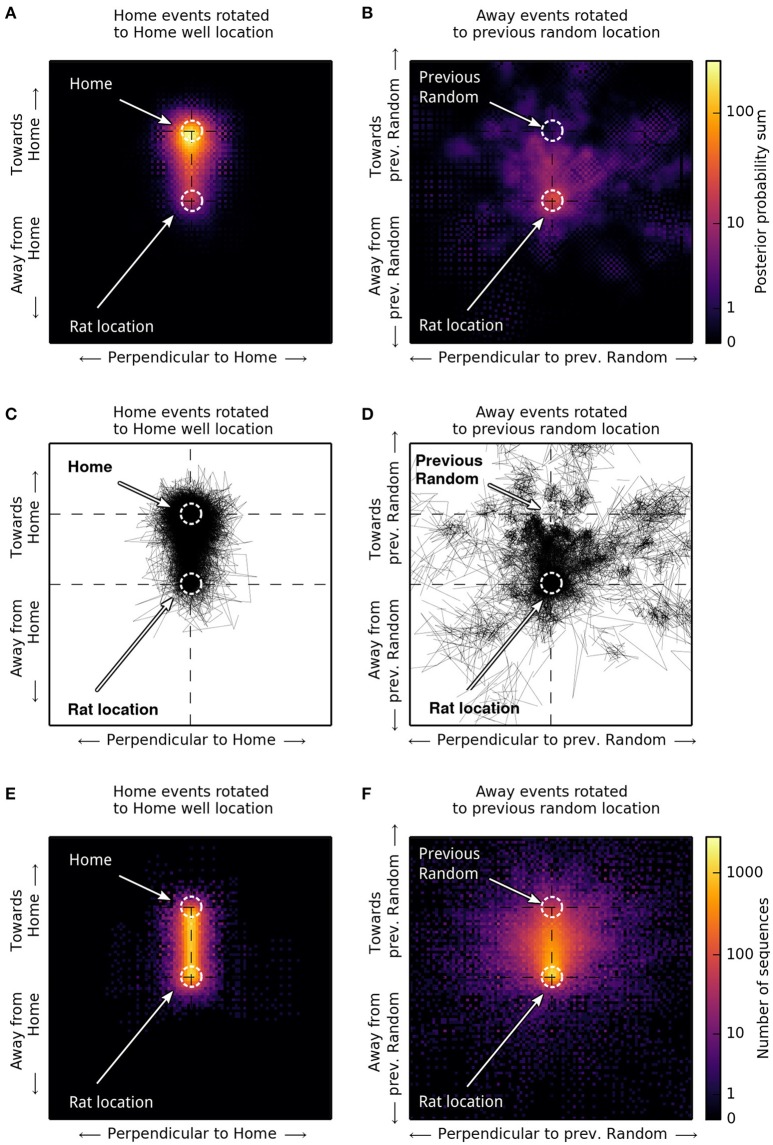
Spatial distribution of sequence trajectories. For comparison with Figure 4A,B in Pfeiffer and Foster (2013), we scaled and rotated sequence trajectories to illustrate their spatial distribution relative to the Home reward location **(A,C,E)** for Home trials, or relative to the previous Random reward location during Random trials **(B,D,F)**. **(A,B)** Posterior probability sums of 480 sequences obtained from 12 networks using Bayesian decoding. **(C,D)** Corresponding decoded sequence trajectories. **(E,F)** Trajectories of 5,760 sequences obtained from all 144 networks using population vector decoding. Sequence trajectories in Home trial phases are strongly biased to proceed toward the Home location **(A,C,E)**. In Random trials, trajectories are biased to proceed toward the previous Random reward location but show a broader spatial distribution **(B,D,F)**.

To illustrate the spatial distribution of sequential activity, we rotated and scaled sequence trajectories relative to a template direction corresponding to a straight-line movement from the simulated rat's position to the active Home location or the previous Random location (Pfeiffer and Foster, 2013). This analysis confirms that place-cell sequences during Home trial phases have a strong tendency to proceed toward the Home feeder location (Figures 8A,C,E). During Away trial phases, place-cell sequences are somewhat biased to proceed toward the previous Random location, but show a broader spatial distribution than in Home trial phases (Figures 8B,D,F). In comparison, Pfeiffer and Foster (2013) have observed a broader spatial distribution of sequence trajectories during Home trial phases, with a somewhat weaker bias toward the Home location than in our model data. Further, the experimental data showed a tendency of trajectories going away from the previous Random location during Random trial phases. These differences can be attributed to our modeling goal of generating sequential activity for goal-prediction with high accuracy, as we have simulated a single place-cell sequence per trial phase for modeling convenience. Additional analyses showed that the pattern of relatively straight movement toward the goal changed toward a broader pattern when spatially correlated noise was added to the synaptic matrix, introducing local excitability biases (Renart et al., 2003). In this setting, mean reward latencies increased, as the goal location was depicted with a lower accuracy (data not shown). We will return to this point in the Discussion.

### 3.4. Quantification of smooth vs. jump-like activity transitions

**Figure 9:**
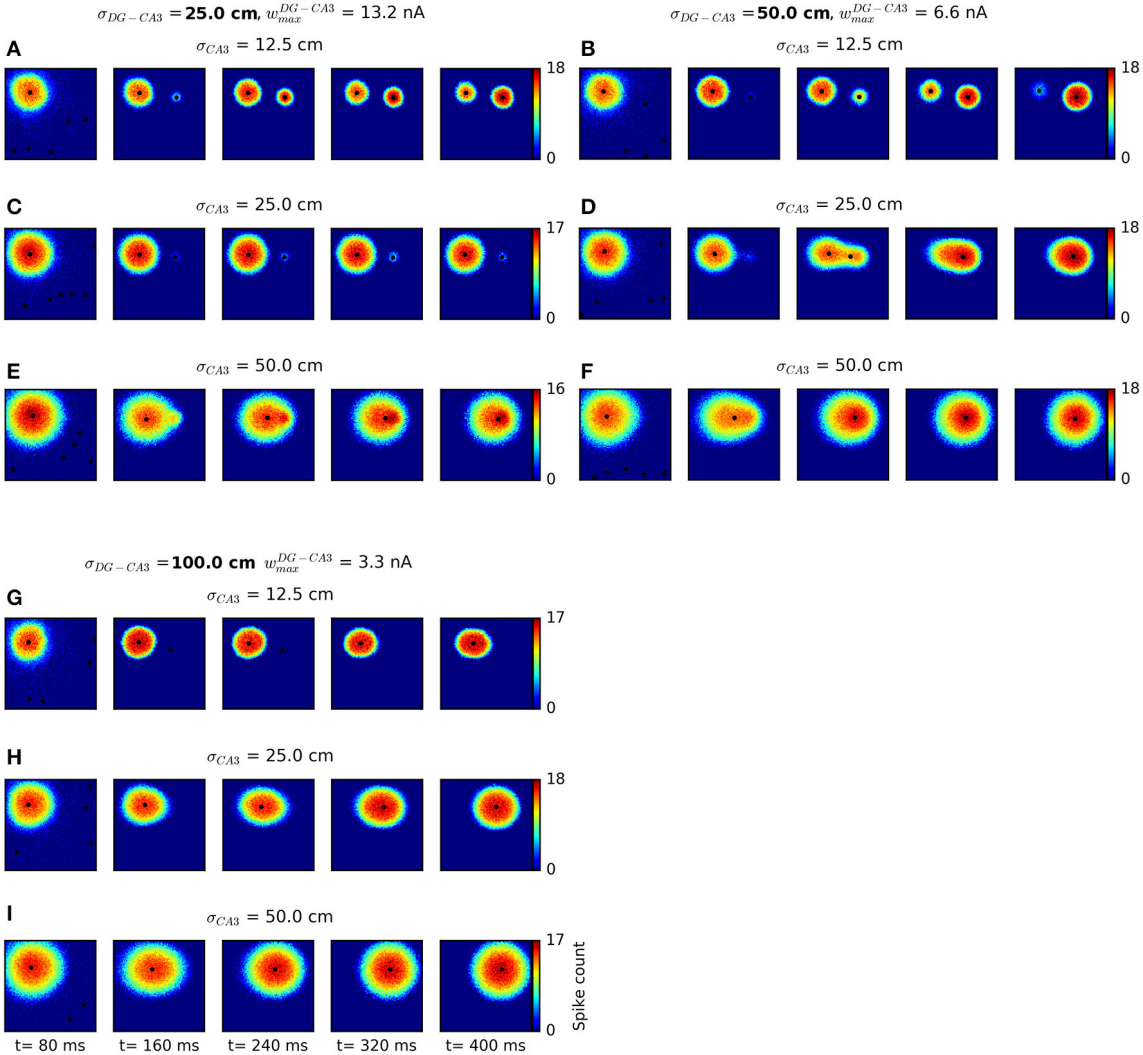
Network dynamics for different connectivity parameter settings. We varied the width of the Gaussian weight profile at the DG-CA3 projection, σ_*DG*−*CA*3_, and the width of the CA3 recurrent excitatory weight profile, σ_*CA*3_. To keep weight sums approximately constant for different connectivity widths, we scaled weight values in proportion to corresponding σ values. Each network configuration was run with initial activation at the top left corner and cortical-DG input near the top right corner of the sheet of cells. Spiking activity is displayed in non-overlapping windows of 80 ms length. Any cluster centers detected by the mean shift clustering algorithm are plotted as black dots. For small bump sizes (resulting from narrower CA3 recurrent weight profiles) and narrower DG-CA3 connectivity profiles, jump-like transitions occur **(A,B,D)**. Gradual transitions are observed for broader DG-CA3 weight profiles **(F–I)**, with broader bumps associated with higher movement speeds. An intermediate regime is also possible **(E)**. In some cases, a weak secondary bump appears without movement of the major bump **(C)**. The default parameters used in behavioral simulations are shown in **(F)**.

To further test the validity of our continuous attractor network approach as a model of hippocampal sequential activity, we examined the conditions under which our model generated smoothly changing activity patterns rather than abrupt transitions. This is particularly warranted as continuous attractor network models are known to give rise to discrete, jump-like movement of the activity peak whenever external stimulation is applied at locations far away from the current activity peak (Ben-Yishai et al., 1995; Degris et al., 2004; Fung et al., 2008), as in the present setting. In recent experiments involving high-density recordings, smooth place-cell sequence trajectories have been discriminated from jump-like sequential activity patterns via a maximum jump size criterion applied to the results of Bayesian decoding: Events in which the distance between the locations decoded from consecutive time windows exceeded a certain threshold were classified as “jump-like” and excluded from further analysis (Pfeiffer and Foster, 2013, 2015). For comparison with these data, we apply similar criteria to the activity patterns generated by our network, in a range of parameter settings. In our model, three main design parameters determine the range at which the transition between smooth and jump-like movement occurs: The widths of the Gaussian weight profiles at both the DG-CA3 and the CA3 recurrent excitatory connections, σ_*DG*−*CA*3_ and σ_*CA*3_, and the strength of DG-CA3 synapses. To analyse bump movement patterns for different parameter settings, we generated nine different network setups and used these to simulate sequential activity with context-to-DG weight matrices stored during the behavioral simulations described above. We used data from twelve out of all 144 networks for this analysis, summing to a total of 480 trials. For an overview of the bump transitions resulting from these different parameter settings, see Figure 9. The default parameters are shown in Figure 9F. In general, broader weight profiles at CA3 recurrent excitatory synapses lead to broader bump sizes, more likely to overlap with a larger range of inputs, in which case gradual movement occurs. On the other hand, broader DG-CA3 weight profiles increase the spatial extent of external inputs at the DG-CA3 pathway without affecting the bump size, and higher DG-CA3 weights are more likely to cause suprathreshold responses of CA3 cells.

**Figure 10:**
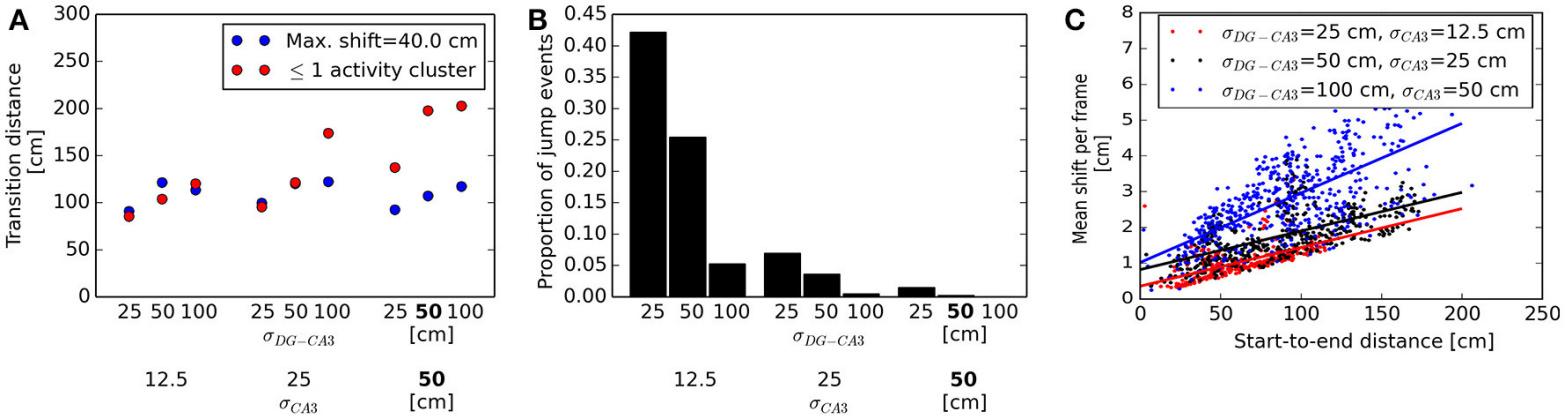
Quantitative analysis of bump dynamics for the range of network parameters shown in Figure 9. **(A)** Transition distances, defined as the start-to-end distance of sequences at which the transition between smooth and jump-like events occurs, across network parameter settings. Results are shown for event classification based on either maximum jump size (blue) or maximum number of activity clusters per frame (red). The two criteria are in good agreement for narrower weight profiles at both the DG-CA3 and the CA3 recurrent connections. For broader weight profiles at both projections, associated with higher average bump speeds, the maximum-shift criterion diverges more strongly from the cluster-based criterion. Default simulation parameters are printed in boldface. **(B)** Proportion of jump-like events across network parameter settings, as determined by the number of activity clusters. Less jump-like events are observed for broader weight profiles at both the DG-CA3 and the CA3 recurrent excitatory connections. **(C)** Relation between bump movement speed and total distance traveled. For each network configuration, a linear fit is shown along with per-trial data. Higher start-to-end distances are associated with faster bump movement for all parameters settings. In addition, larger bump widths are associated with higher overall speed.

For a quantitative analysis of the parameter settings shown in Figure 9, we classified sequences as “non-jump” or “jump” events and determined the range at which the transition between the two regimes occurs. We defined this transition distance as the value *d* which best separated the distributions of start-to-end distances of non-jump and jump-like sequences, such that a proportion of 1−α of jump-like sequences had a start-to-end distance greater than *d*, and an equal proportion of non-jump sequences had a start-to-end distance less than *d*. Resulting α values ranged between zero and 0.3. Using a maximum jump size criterion for event classification revealed transition distances between approx. 90 and 200 cm (a case in which no jump transitions were detected), with broader DG-CA3 weight profiles associated with larger transition distances (Figure 10A). This criterion did not indicate a consistent effect of the width of the CA3 recurrent weight profile, owing to the different bump speeds associated to broader vs. narrower bump widths (cf. Figure 9G–I). We therefore considered another criterion based on a cluster analysis of the spatial distribution of activity, independent of bump speed: Events in which more than one activity cluster was detected in any of the decoding frames were considered as jump-like transitions (see Methods for details). For narrower profiles at both the DG-CA3 and the CA3 recurrent connections, the transition distances specified by this criterion were in good agreement with those previously determined by the maximum jump size criterion. For broader weight profiles at both connections, the cluster-based criterion resulted in higher transition distances than the maximum jump size criterion (Figure 10A). As the cluster-based discrimination was more consistent across different parameters, we determined the proportion of jump-like relative to smooth trajectory events based on this criterion, ranging from approx. 40% for narrow weight profiles at both the DG-CA3 and the CA3 recurrent projections to 0% for broader profiles at both projections (Figure 10B). For comparison, Pfeiffer and Foster (2013) have reported percentages of confirmed non-jump events in the range of 25–44% of all candidate SWR events. Finally, we found that the speed of bump movement in non-jump events, as measured by its mean displacement across decoding frames, is a monotonically increasing function of the start-to-end distance (Figure 10C). This prediction may be directly tested experimentally. In addition, larger bump widths lead to higher velocities in our simulations.

To summarize, we can quantify the proportion of jump-like events relative to smooth events and confirm that the vast majority of the simulated events using our default parameters are smooth transitions, with a mean speed dependent on the total distance traveled.

### 3.5. Temporal profile of population dynamics

**Figure 11:**
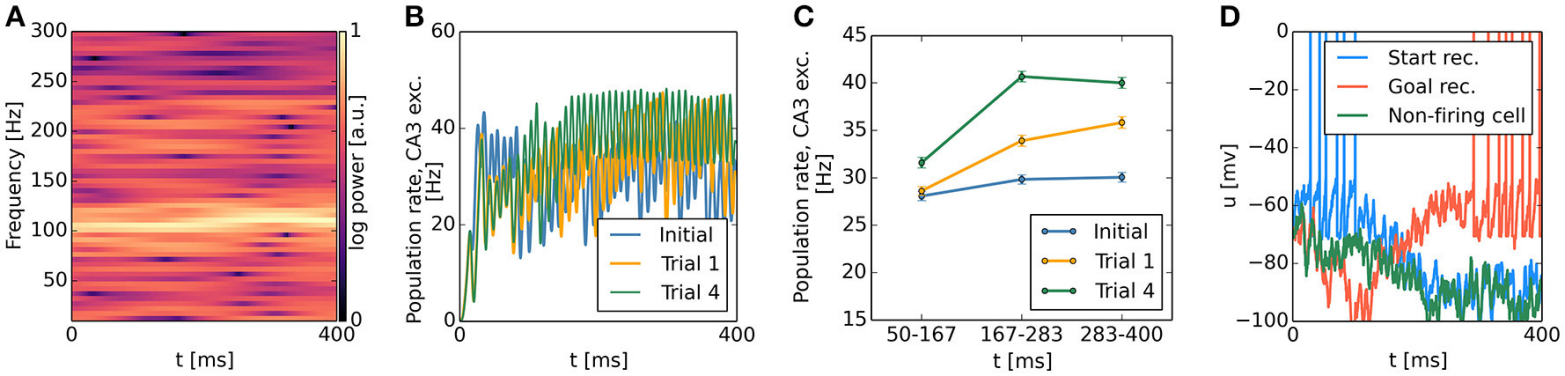
CA3 population activity and subthreshold membrane potential dynamics during sequence generation. **(A)** Representative spectrogram of CA3 excitatory population rates, showing increased power in the 100–250 Hz ripple band. FFT spectra were computed with a Hanning window of size 1024, advanced in increments of 2 ms. **(B)** Time course of CA3 excitatory population rates during sequence generation before the first trial, after the first trial, and after the fourth trial. Population rates were smoothed with a Gaussian kernel of 2 ms width. **(C)** Time-average (mean ± s.e.m.) of population rates shown in **(B)** for early, middle, and late phases of each sequence. Before learning, firing rates remain approximately constant. After the first rewarded trial, higher population rates are observed in the middle and last third of a sequence relative to the first third. This effect becomes more pronounced after additional trials. **(D)** Representative membrane potential traces of CA3 place cells with place field locations near the start and end points of the sequence as well as a silent cell with a place field not overlapping with the sequence trajectory. Cells participating in the bump trajectory show ramp-like dynamics, with gradual ramping depolarization for cells firing late in the sequence, and gradual ramping hyperpolarization for cells firing early in the sequence. Silent cells show increasing hyperpolarization across a single sequence.

Our continuous attractor network model biased by spatially localized inputs, which originate from a contextual goal memory signal, predicts a specific temporal profile of population activity during single SWR sequences. We first analyzed the spectral content of the population firing rates of CA3 excitatory cells and observed strong periodicity in the 100–250 Hz ripple band (Figure 11A), caused by highly synchronous oscillatory activity in CA3 inhibitory cells (Figures 6A,B). While CA3 population firing rates were strongly oscillatory, time-averaging across the first, second and last third of simulated sequences revealed an activity increase in trials following reward-based learning, but not in the initial condition: The activity of CA3 excitatory cells grows as the attractor bump gradually moves toward those cells receiving additional subthreshold excitation (Figures 11B,C). A similar pattern can be observed in the subthreshold membrane potential dynamics of CA3 place cells. Cells with a place field near the goal, where the sequence trajectory ends, show a gradual ramp-like depolarization over the time course of the sequence. Cells firing early in the sequence show gradual hyperpolarization once they have stopped firing. Finally, the membrane potential of place cells that remain silent throughout an SWR event shows an increasing degree of hyperpolarization over the time course of a simulated SWR event owing to increasing CA3 population rates (Figure 11D). This prediction can be directly tested experimentally with intracellular recording techniques. To our knowledge, available intracellular data from place cells during SWRs do not address this question (English et al., 2014).

## 4. Discussion

We have presented a model that explains the learning and recall of goal locations and the generation of place-cell sequences as interdependent processes. The model generates goal-predictive sequential activity of place cells, including trajectories not previously visited, as an effect of continuous attractor network dynamics biased by memory traces at cortico-hippocampal synapses. Importantly, this account of sequence generation does not depend on the storage and recall of specific trajectories. The resulting place-cell sequences support efficient goal navigation in a memory-guided decision-making task, comparable to animal performance in the same task (Pfeiffer and Foster, 2013).

### 4.1. Relation to experimental data

Our model uses the memory-guided decision-making task described by Pfeiffer and Foster (2013) to demonstrate the utility of goal-directed sequential activity in tasks requiring high behavioral flexibility. However, Pfeiffer and Foster (2013), observed a diversity in sequential activity patterns that occurred in close temporal proximity, whereas we simulated only a single sequence. While these experimental data show a bias for sequence trajectories toward the goal location, typically several place-cell sequences progressing into different directions were observed prior to navigation toward the remembered goal location, indicating a less direct involvement in navigation than we assumed here for simplicity. This aspect is highly relevant to the functional interpretation of awake sequential activity. A prominent model suggests that several SWR-associated place-cell sequences can act as “exploratory” sequences for evaluation of competing options (Carr et al., 2011; Erdem and Hasselmo, 2012; van der Meer et al., 2012; Pezzulo et al., 2014). In line with this view, the different trajectories associated with multiple place-cell sequences may originate from a form of “mental navigation” along several directions of imagined movement (e.g., Byrne et al., 2007), potentially caused by grid cell activity driving place-cell sequences (Erdem and Hasselmo, 2012). However, recent parallel recordings of hippocampal place cells and grid cells in the medial entorhinal cortex (MEC) during sleep-associated SWR sequences have found that grid cell representations from MEC deep layers were briefly delayed relative to place cells (Olafsdóttir et al., 2016), and that MEC superficial layers generate replay sequences independently of the hippocampus (O'Neill et al., 2017). These results challenge the view that place-cell sequences are predominantly driven by grid cell activity, highlighting the need for other mechanisms of sequence generation.

We have departed from the following alternative hypothesis: Rather than performing a form of mental navigation defined by a particular direction, our method of sequence generation requires defining a “recall context,” here defined by the reward contingency (i.e., reward placed at the Home location vs. placed at a random location). This approach is inspired by the suggestion that prefrontal representations, influenced by context and previous outcomes, may exert a bias on hippocampal recall activity, potentially signaling optimal responses (Euston et al., 2012; Preston and Eichenbaum, 2013). In principle, our model allows to generate a larger number of place-cell sequences by increasing the number of “recall context” populations, perhaps corresponding to different hypotheses of where the goal location may be located. However, modeling a functional role of different sequence trajectories would require adding an evaluation component downstream of the hippocampus, such as the ventral striatum. Addressing the origins of the observed variability in sequence trajectories remains a key topic for future studies.

The present study focuses on the generation of forward-ordered sequential activity originating at the animal's current location within an open-field maze, where hippocampal place cells show no directional selectivity. By contrast, when rats shuttle between two feeders placed at the ends of a linear track, place cells in DG and CA1 are typically active only in one of the two movement directions (Gothard et al., 2001), which makes it possible to classify place-cell sequences as “forward” or “reverse” replay. In addition, CA3 place cells tend to fire at different locations depending on running direction (Miao et al., 2015). We briefly discuss how our model may be extended to generate reverse replay sequences. First, a directional selectivity of place cells can be obtained by incorporating a multi-chart network structure, similar to the model by Azizi et al. (2013): Two “directional charts” can be formed from place cells in DG and CA3 by independently assigning two place field center locations to each cell (one for each movement direction). To ensure that an attractor bump can form in each chart, the strength of recurrent synapses between CA3 place cells is configured as a Gaussian function of distance within each chart. Switching between charts may be based on both visual landmark cues and proprioceptive signals (e.g., a turn). Activity of the two cortical context populations will code for approaching a specific feeder in the linear track setting, and switching between these representations is likely triggered by reward delivery. Importantly, reward associations at context-to-DG synapses may extend to the “new” place cell chart (active after leaving the reward location) if traces of the dopaminergic reward signal (or, alternatively, synaptic traces of recent presynaptic cortical context activity) persist beyond context remapping. Once reward associations have formed, the temporal order of remapping determines whether sequential activity will occur in a forward or reverse direction: Whenever contextual remapping takes place before the switch in place cell charts occurs, the secondary reward association will drive SWR-associated place cell activity across the previously active chart toward the opposite-end feeder, resulting in reverse replay. However, if contextual remapping takes place after chart switching, forward replay activity will be generated.

### 4.2. Relationship to existing models

Existing models of place-cell sequences can be broadly grouped into three categories. First, sequence learning models assume unidirectional strengthening of CA3 recurrent synapses during repeated traversals of a maze segment (Jensen and Lisman, 1996; Redish and Touretzky, 1998; Molter et al., 2007; Bush et al., 2010; see also Levy, 1996; Chenkov et al., 2017). These models explain the generation of forward replay sequences by strong recurrent weights during recall. An exception is the model by Jahnke et al. (2015) in which synchronous inputs trigger replay of learned sequences owing to supralinear summation of dendritic inputs. The main difference with our approach is that specific trajectories are encoded in CA3 recurrent synapses in these models. While explaining the generation of replay sequences replicating trajectories stored during navigation in track-like mazes, including large environments in which extended replay across several SWR events has been observed (Davidson et al., 2009), it is not obvious how these models may generalize to open-field navigation tasks. Second, continuous attractor models of place-cell sequences generate spatially random sequence trajectories in the presence of firing-rate adaptation (Hopfield, 2010; Azizi et al., 2013), spike threshold adaptation (Itskov et al., 2011), or short-term plasticity (Romani and Tsodyks, 2014). In these models, contrary to our approach, external input to the CA3 network serves mainly as background excitation and is therefore assumed as spatially homogeneous. This class of models is based on earlier work by Muller et al. (1996) and Samsonovich and McNaughton (1997). Finally, models based on “lingering place-cell excitability” (Foster and Wilson, 2006; Diba and Buzsáki, 2007; Atherton et al., 2015) propose that reverse replay sequences originate from an interplay between spatially tuned inputs and a gradually decreasing level of inhibition. A recent conceptual proposal provides an integrative view by suggesting that each of the different mechanisms of sequence generation may operate in a distinct behavioral state, and that their coordination is mediated by neuromodulators such as acetylcholine and dopamine (Atherton et al., 2015).

A few computational models account for the generation of place-cell sequences with a functional role in goal-directed behavior. The model of Erdem and Hasselmo (2012) is based on linear exploratory “look-ahead probe” activity driven by grid cells, and its performance in finding a known goal location is demonstrated in a variety of open-field and structured mazes. However, as discussed above, the assumption of grid cells driving place-cell sequences has been questioned by recent experimental data (Olafsdóttir et al., 2016; O'Neill et al., 2017). In another model based on a more abstract statistical approach, Penny et al. (2013) have shown that goal-predictive sequential activity can be replicated by probabilistic inference processes. Moreover, Corneil and Gerstner (2015) have proposed a model in which a theoretically derived “successor representation” is approximated by a continuous attractor network to generate goal-directed sequential activity. Their model is conceptually similar to our study, but shows a number of differences worth highlighting. Corneil and Gerstner (2015) have combined a mathematical analysis with a relatively abstract network implementation and presented a qualitative prediction in terms of the effect of place field sizes, which was directly linked to the attractor bump size in their network. By contrast, our approach using a large-scale spiking network with physiologically interpretable parameters integrates reward-based synaptic plasticity as a model of goal learning and allows detailed comparisons of the network's spiking dynamics to experimental data. In addition, the present study offers quantitative measures of the transition between smooth and jump-like activity patterns as a function of the model parameters.

Previous models of spatial learning differ in the way they can deal with changing goals. In a number of models, an association between place cell activity and a direction toward a goal location is learned. A distinct place cell map representation for each goal is required both in these models and in another model in which place cells cluster near a goal location to create a gradient to be followed (Gerstner and Abbott, 1997; Vasilaki et al., 2009; Clearwater and Bilkey, 2012). In the Burgess et al. (1994) model, the direction toward the goal is represented by a set of “goal cells”. Here, multiple goals could be represented by different sets of goal cells. Considering the range of goal-finding, the size of the largest place fields determines performance in the Burgess et al. (1994) model. In our model, the range of goal-finding depends on network activity levels during simulated SWRs, as sequential activity requires the attractor bump to overlap with potentiated context-to-DG weights convoluted by the DG-CA3 connectivity pattern. Finally, the model proposed by Foster et al. (2000) uses a learned spatial coordinate function to derive abstract “goal coordinates”, which can be flexibly updated. Our model, by contrast, implements the flexible contextual encoding and recall of goal locations as a neural-level mechanism.

### 4.3. Physiological evidence for the model mechanisms

In its essence, the functioning of our model depends on continuous attractor dynamics combined with external inputs modifiable by goal learning. We have hypothesized that these functions may be mapped onto a cortico-DG-CA3 pathway, and we briefly review relevant experimental evidence. First, contextual biases in cortical activity are key to the context-specific learning of goal locations, which in turn allows to bias the content of place-cell sequences in our model. Representations of task phase or temporal context have been reported in prefrontal areas (Hyman et al., 2012; Waskom et al., 2014). Several pathways may transmit these contextual codes from the prefrontal cortex to the hippocampus. Recent studies observed projections from anterior cingulate cortex to terminate in the CA3 and CA1 subfields, but not in the DG (Ito et al., 2015; Rajasethupathy et al., 2015). However, prefrontal areas project to the perirhinal and lateral entorhinal cortices (Apergis-Schoute and Paré, 2006), which innervate the dentate gyrus, and it has been suggested that this pathway may allow prefrontal control over memory retrieval (Preston and Eichenbaum, 2013), consistent with our assumptions.

We have assumed that reward-dependent plasticity is expressed at cortico-DG synapses, as the dentate gyrus, but not CA3, receives noradrenergic and dopaminergic innervation (Amaral and Lavenex, 2006), associated with modulation of plasticity (Seidenbecher et al., 1997; Manahan-Vaughan and Kulla, 2003; Straube et al., 2003; Hamilton et al., 2010; Yang and Dani, 2014; Hansen and Manahan-Vaughan, 2015; Takeuchi et al., 2016). Noradrenergic and dopaminergic terminals are also present in area CA1 (Amaral and Lavenex, 2006). Considering that continuous attractor network dynamics do not require recurrent synaptic connectivity as found in CA3, but can also be based on cross-inhibition (Song and Wang, 2005), this suggests that the functioning of our model may alternatively be mapped onto the direct PFC-CA1 pathway.

We have hypothesized that DG activity can bias the content of hippocampal sequential activity, an assumption which, to our knowledge, has not yet been experimentally tested. Available data do support an influence of dentate gyrus activity both on the occurrence probability of SWR episodes and on CA3 slow gamma activity. During slow-wave sleep, Sullivan et al. (2011) observed that ripple events occurred more frequently in the 250 ms following “UP-DOWN” transitions (i.e., from states of average to high DG activity to states of low DG activity) than in the 250 ms preceding those transitions. However, the same study also noted that the relative timing of peak SWR activity between CA1 and DG was inconsistent across animals, indicating that caution must be applied to interpretations regarding causality. During awake behavior, Hsiao et al. (2016) have investigated the relation between gamma rhythmic activity in DG and CA3. Their study reported directional causal influences of DG slow gamma on CA3 slow gamma, measured by Granger causality analysis, and phase-locking of DG place-cell spikes to CA3 slow gamma, indicating that the influence of DG on CA3 may rely on direct excitatory synaptic transmission from DG to CA3. Further, increased levels of slow gamma activity have been observed during awake SWR episodes (Carr et al., 2012). Finally, in a radial maze task, Sasaki et al. (2014) have observed that awake SWR events occurring at reward sites were absent in DG-lesioned rats, while ripple events occurring at the maze stem were unaffected. Taken together, these findings suggest that DG activity can influence CA3 activity during sharp-wave ripples.

Our proposed role for CA3 in the recall of goal locations is consistent with the observation of deficits in spatial memory retrieval following lesions of the CA3 subfield (Brun et al., 2002). We have further hypothesized that the recall dynamics in CA3 can be modeled by continuous attractor network dynamics. Although this assumption is shared by a number of previous models as discussed above, it has been noted that testing the continuous attractor hypothesis experimentally has proved challenging (Knierim and Zhang, 2012). Recently, Pfeiffer and Foster (2015) have argued for the presence of discrete attractor (or autoassociative) dynamics in place-cell sequences, as they found the step sizes of decoded sequence trajectories to be temporally correlated with slow-gamma oscillations, consistent with step-like transitions between attractor patterns. However, we note that discrete, pulse-like bursts of oscillatory activity were also observed in a model in which a graded, rather than discrete, structure of recurrent connectivity between place cells emerged by sequence learning (Jahnke et al., 2015), suggesting that it may prove difficult to accurately discriminate between a spatially discrete structure of place-cell sequences and a predominantly temporal discretization resulting from strong population oscillations.

### 4.4. Predictions

A key prediction of our model is that DG cells with place fields near the goal location should display sustained firing throughout place-cell sequences which proceed toward that goal. This contrasts with the sequential activity patterns observed in CA1 place cells and, recently, MEC grid cells (O'Neill et al., 2017). To our knowledge, the locations represented in DG and CA3 activity during SWR events have not yet been specifically examined. Interestingly, sustained representations in perirhinal neurons were recently observed during a cued spatial decision-making task (Bos et al., 2017). In addition, our model shares several predictions with other continuous attractor network models biased by external inputs. We have shown that the propagation speed of place-cell sequences is a function of their start-to-end distance in our model. Furthermore, ramp-like temporal profiles are observed both in CA3 population rates and in subthreshold membrane potential dynamics over the time course of single sequences. These predictions can be directly tested experimentally.

In addition, our model predicts that both sequence generation and flexible goal navigation will be impaired if any of its critical components – contextual coding, reward-based plasticity and continuous attractor dynamics – are interfered with. This relates to experimental studies in which multi-stage synaptic transmission between prefrontal areas and dentate granule cells (e.g., via perirhinal and lateral entorhinal cortices) has been functionally inactivated (Lu et al., 2013), or involving NMDA receptor deletion in the dentate gyrus (McHugh et al., 2007; Bannerman et al., 2012). To our knowledge, the potential effect of these manipulations on SWR-associated place cell sequences has not yet been investigated. Moreover, Suh et al. (2013) have studied hippocampal sequential activity in mice lacking the *fCNB1* gene in CA1 and the dentate gyrus. While this manipulation has been shown to affect plasticity at CA3-CA1 synapses, accompanied by deficits in tasks involving changing goals (Zeng et al., 2001), it likely causes similar effects at the lateral perforant path synapses onto DG granule cells. In the framework of our model, the impairments in SWR-associated replay reported by Suh et al. (2013) can be explained by impairments in plasticity at DG inputs.

### 4.5. Limitations

As this study focuses on the potential role of plasticity at DG inputs in the generation of place-cell sequences, we have assumed that CA3 recurrent synapses are non-plastic and show a symmetric, map-like weight profile. By contrast, several experimental results obtained in track-like environments have provided evidence for experience-dependent asymmetric potentiation of CA3 recurrent synapses, much like a sequence learning process (Mehta et al., 2000; Ekstrom et al., 2001; Lee et al., 2004). In our view, these contrasting hypotheses about the weight structure of CA3 recurrent synapses can be reconciled in the following way: It has been shown that triplet-based spike timing-dependent synaptic plasticity (STDP) rules (Pfister and Gerstner, 2006, see also Sjöström et al., 2001) are capable of generating asymmetric weight profiles in the presence of systematic timing differences between neurons, and a symmetric weight structure in the presence of rate correlations without a temporal code (Bush et al., 2010; Clopath et al., 2010). We therefore hypothesize that in open field navigation, CA3 recurrent weights will be symmetric, reflecting the rate correlations between overlapping place cells in the absence of any specific directional bias during running. In track-based navigation tasks, however, an asymmetric profile of CA3 recurrent weights is likely to emerge given the highly sequential structure of the task. In our interpretation, these task-specific weight profiles may affect the spatiotemporal dynamics of sequence trajectories: The finding of an approximately constant propagation speed of place-cell sequences across a large track-like environment Davidson et al. (2009) is potentially consistent with a sequence recall process, while our simulation results show a distance-dependent speed profile of sequence trajectories. Further, we hypothesize that our model may be generalized to episodic memory recall in the non-spatial domain, e.g., odor sequences (DeVito and Eichenbaum, 2011) or lists of arbitrary items (Kahana, 1996) if plasticity at CA3 recurrent synapses is incorporated.

For modeling convenience, we have incorporated several idealizations: As the focus on this work is on the dynamics of a recall process biased by contextual input, these context representations are hard-wired in our model. However, we have recently demonstrated how prefrontal category representations can emerge in a rewarded task (Villagrasa et al., 2016), a mechanism which will be integrated into this model at a later stage. For simplicity, we have considered DG cells with a single place field, although multiple place fields have been reported for DG granule cells (Jung and McNaughton, 1996; Leutgeb et al., 2007; Neunuebel and Knierim, 2012). Whether this has any implications for the mechanism proposed here requires further investigation. Furthermore, we have modeled hippocampal subfield CA3 but not CA1, from which most experimental recordings of hippocampal sequential activity are obtained. Previous studies have shown that sequences in area CA1 can be inherited from area CA3 (Itskov et al., 2011; Jaramillo et al., 2014), and we therefore assume that if a CA1 layer was added to our network model, it would show sequential activity with a similar structure as in our CA3 layer. We have not explicitly modeled the mechanism by which the CA3 network is initialized to represent the animal's current position. In models of look-ahead (or “mind-travel”), during movement-related theta rhythm, a hippocampal representation of current position is generated based on entorhinal grid cell inputs (Sanders et al., 2015). During SWR-related place-cell sequences, this mechanism may depend on the CA2 region of the hippocampus (Kay et al., 2016, see also Oliva et al., 2016). Finally, it has been suggested that information exchange between hippocampus and mPFC may take place in both directions (Euston et al., 2012; Jadhav et al., 2012; Preston and Eichenbaum, 2013). Investigating the influence of hippocampal output on neocortical representations is a key challenge for future research.

### 4.6. Summary

To conclude, we have shown how goal-anticipating place-cell sequences may originate from the combined effects of neocortical contextual coding, goal memory formation at cortico-hippocampal synapses, and continuous attractor dynamics, without storage of individual trajectories or drive by virtual self-motion signals. We have demonstrated the utility of these sequences, which include novel trajectories across familiar terrain, in a memory-guided navigation task. In the complex picture of different patterns of SWR-associated place-cell sequences which has emerged over the past two decades, this study adds a piece to the mosaic of multiple mechanisms which collectively may explain the variety of hippocampal sequential activity.

## Author contributions

Designed research: LG, JV, and FH. Guided research: JV and FH. Performed research: LG. Writing: LG, JV, and FH.

### Conflict of interest statement

The authors declare that the research was conducted in the absence of any commercial or financial relationships that could be construed as a potential conflict of interest.
